# The resurrection of RIP kinase 1 as an early cell death checkpoint regulator—a potential target for therapy in the necroptosis era

**DOI:** 10.1038/s12276-022-00847-4

**Published:** 2022-09-28

**Authors:** Eunjin Ju, Kyeong Ah Park, Han-Ming Shen, Gang Min Hur

**Affiliations:** 1 Department of Pharmacology and Department of Medical Science, College of Medicine, Chungnam National University, Daejeon, 35015 Republic of Korea; 2 Faculty of Health Sciences, Ministry of Education Frontiers Science Center for Precision Oncology, University of Macau, Macau, China

**Keywords:** Apoptosis, Necroptosis, Checkpoint signalling

## Abstract

Receptor-interacting serine threonine protein kinase 1 (RIPK1) has emerged as a central molecular switch in controlling the balance between cell survival and cell death. The pro-survival role of RIPK1 in maintaining cell survival is achieved via its ability to induce NF-κB-dependent expression of anti-apoptotic genes. However, recent advances have identified the pro-death function of RIPK1: posttranslational modifications of RIPK1 in the tumor necrosis factor receptor 1 (TNFR1)-associated complex-I, in the cytosolic complex-IIb or in necrosomes regulate the cytotoxic potential of RIPK1, forming an early cell death checkpoint. Since the kinase activity of RIPK1 is indispensable in RIPK3- and MLKL-mediated necroptosis induction, while it is dispensable in apoptosis, a better understanding of this early cell death checkpoint via RIPK1 might lead to new insights into the molecular mechanisms controlling both apoptotic and necroptotic modes of cell death and help develop novel therapeutic approaches for cancer. Here, we present an emerging view of the regulatory mechanisms for RIPK1 activity, especially with respect to the early cell death checkpoint. We also discuss the impact of dysregulated RIPK1 activity in pathophysiological settings and highlight its therapeutic potential in treating human diseases.

## Cell biology: a protein controlling whether cells live or die

Improved understanding of the molecular mechanisms that allow a protein to control the balance between cell survival or early death could reveal new approaches to treating conditions including chronic inflammatory disease and cancer. Gang Min Hur and colleagues at Chungnam National University in Daejeon, South Korea, with Han-Ming Shen at the University of Macau in China, review emerging evidence about how the protein called receptor-interacting serine/threonine-protein kinase 1 (RIPK1) influences whether cells move towards death or survival at a key ‘checkpoint’ in cell development. Cells can undergo a natural process of programmed cell death called apoptosis, die abnormally in a disease process called necroptosis, or survive. RIPK1 appears able to influence which path is chosen depending on which genes it regulates and which proteins it interacts with. Many details are still unclear, and need further investigation.

## Introduction

Apoptosis is considered to be an evolutionarily conserved cell death process for normal development and homeostasis, whereas necrosis is historically considered to be an accidental, uncontrolled and passive cell death phenomenon evoked by environmental perturbations^1,2^. The discovery of the caspase homolog CED-3 and the Apaf-1 homolog CDD-4 in the nematode C. elegans led to the establishment of apoptosis as a regulated cell death pathway^3,4^. The apoptosis pathway in mammals is characterized by the activation of a series of caspase cascades that ensure cell death occurs in a controlled manner^5,6^. Consequently, caspase inhibition is capable of protecting cells against either extrinsic or intrinsic cytotoxic damage. However, in certain types of cells, genetic or pharmacological inhibition of caspases is unable to block cell death upon the ligation of death receptors, including the tumor necrosis factor receptor 1 (TNFR1), but rather facilitates the necrotic cell death process^7–9^. The molecular mechanism of such paradoxical death receptor-mediated necrotic cell death has long remained unknown. It is now becoming increasingly clear that receptor-interacting protein kinase 1 (RIPK1) is a key player in the control of this phenomenon, as RIPK1 is required to execute necrosis in a caspase-independent manner. Importantly, RIPK1 acts upstream of another key protein kinase, RIPK3, by forming a microfilament-like complex called the necrosome, leading to the activation of the pro-necrotic enzyme mixed lineage kinase domain-like pseudokinase (MLKL), which causes cell membrane disruption and ultimately results in programmed necrosis, which is called necroptosis, an alternative pathway for programmed cell death (PCD)^10,11^. Furthermore, a recent structural study indicated that activation of RIPK1 is required for the induction of both RIPK1/RIPK3 hetero- and RIPK3 homo-oligomerization in the early stages of necroptosis, which leads to an ordered, helical formation of necrosome rods^12^. Subsequently, such a configuration for necrosome-associated RIPK1/RIPK3 oligomers functions to facilitate MLKL oligomerization and execute necroptosis^12^, indicating that RIPK1 indeed functions as a signaling hub in RIPK3-dependent necroptotic cell death.

**Fig. 1:**
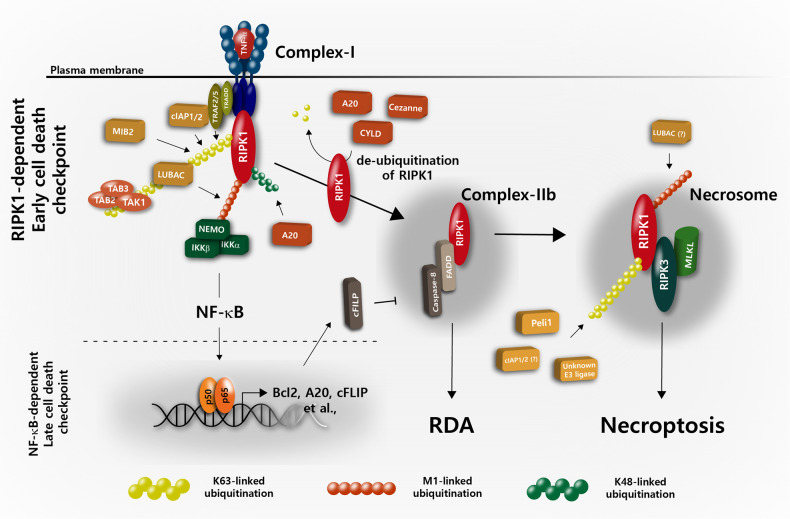
Regulation of the RIPK1-mediated early cell death checkpoint by ubiquitination. Ligation of TNFR1 triggers the formation of the primary TNFR1 complex, referred to as “complex-I,” by the recruitment of TRADD and RIPK1, which in turn recruits several E3 ubiquitin ligases. The early cell death checkpoint is initiated by the conjugation of K63 polyUb chains or M1-linked linear Ub chains on RIPK1 by TRAF2/5, cIAP1/2 and MIB2 or LUBAC. The ubiquitinated RIPK1 within complex-I keeps RIPK1 in a survival mode and prevents the formation of a secondary cytoplasmic complex, referred to as “complex-IIb,” in a gene transcription-independent manner. This process also allows docking of TAK1 with TAB2 or TAB3 as well as of the IKK complex (IKK-α/-β/NEMO) to activate NF-κB, which functions as a late cell death checkpoint by the transcriptional induction of survival genes, including Bcl2, A20 and c-FLIP. Deubiquitination of RIPK1 by either A20, CYLD and Cezanne or the inhibition of E3 ubiquitin ligases promotes the sequential formation of the pro-apoptotic complex-IIb consisting of RIPK1, FADD and caspase-8 to mediate RIPK1-dependent apoptosis (RDA). Alternatively, in the absence of apoptosis, RIPK1 interacts with RIPK3 and MLKL to form necrosomes, which elicits downstream events for necroptosis. Under necroptotic conditions, several E3 Ub ligases, such as PELI1, cIAP1/2 and LUBAC, are also proposed to target cytosolic RIPK1 within complex-IIb or necrosomes for ubiquitination. Abbreviations: TNFR1, tumor necrosis factor receptor 1; TRADD, TNFR1-associated death domain protein; RIPK1/3, receptor-interacting serine threonine protein kinase 1/3; TRAF2/5, TNF receptor-associated Factor 2/5; cIAP1/2, cellular inhibitor of apoptosis 1/2; MIB2, Mind Bomb-2; LUBAC, linear ubiquitin chain assembly complex; TAK1, transforming growth factor β activated kinase 1; TAB2, TAK1-binding protein 2, IKK Inhibitory kappa B kinase, NF-κB nuclear factor-kappa B, Bcl2 B-cell lymphoma 2, c-FLIP cellular FLICE-inhibitory protein, CYLD cylindromatosis lysine 63 deubiquitinase, FADD Fas-associated death domain, MLKL mixed lineage kinase domain-like pseudokinase, PELI1, Pellino 1.

RIPK1 was initially identified as an adapter protein that interacts with the death domain (DD) of CD95 or TNFR1 via a homotypic DD-DD interaction that leads to a plasma membrane-bound death receptor (DR) signaling complex (also known as complex-I)^13,14^. Subsequently, complex-I internalizes and dissociates from the plasma membrane and recruits the Fas-associated death domain (FADD) and caspase-8 to form a cytoplasmic death-inducing signaling complex (DISC, also known as complex-IIa) that elicits characteristic apoptotic cell death^13,14^. Subsequently, another protein interaction motif in RIPK1, termed RIP homotypic interaction motif (RHIM), was identified, and this motif mediates interactions with RIPK3 to trigger necroptosis^15,16^. In addition, RIPK1 interacts with numerous adapter proteins and with the inhibitory κB kinase (IKK) complex, resulting in the activation of nuclear factor-κB (NF-κB)^17–19^. This process then drives the transcriptional induction of prosurvival genes such as Bcl2, A20 and cFLIP by an NF-κB-dependent process that protects against apoptosis, and this is referred to as the NF-κB-dependent late cell death checkpoint (also called the transcription-dependent cell death checkpoint^20,21^ (Fig. 1)). Previous studies have demonstrated that NF-κB-dependent transcription of pro-survival genes regulates apoptotic signals in multiple ways. For instance, cFLIP, a catalytically inactive caspase-8 homolog, interacts with procaspase-8 and prevents the processing of caspase-8 by counteracting the cytotoxic activity of complex-IIa^22,23^. Thus, it is believed that NF-κB-dependent transcription of c-FLIP expression keeps the sublethal activation of DISC in check, thus preventing DR-mediated apoptosis. On the other hand, other NF-κB-inducible genes, such as Bcl2 and Bcl-_XL,_ also function as late cell death checkpoints by regulating the mitochondria-mediated apoptotic pathway^24^. Thus, RIPK1 has long been studied as a key molecule in determining cell fate in the field of NF-κB signaling. However, recent studies have reported that the posttranslational modifications (PTMs) of RIPK1 repress the cytotoxic potential of RIPK1 in complex-I and thereby protect cells from cell death by counteracting the assembly of complex-IIb (RIPK1-FADD-caspase-8), which is independent of the transcriptional activation of NF-κB and is referred to as the RIPK1-dependent early cell death checkpoint^25–27^ (Fig. 1). Given that the cytotoxic potential of RIPK1 is essential for engaging the necroptotic cell death machinery via RIPK3^28^, insights into this regulation have not only led to an improved understanding of a fundamental signaling node in necroptosis but also offered an opportunity to develop new therapeutic approaches in necroptosis-associated human diseases^29,30^. Since transcription-dependent cell death regulation by NF-κB activation has been discussed extensively elsewhere^29,31^, in this review, we mainly focus on recent developments in the study of the early cell death checkpoint where RIPK1 acts to regulate two PCD pathways in the forms of apoptosis and necroptosis.

## Regulation of the cytotoxic potential of RIPK1 by ubiquitination in complex-I

RIPK1 is well known to play a key role in TNF signaling. Within seconds of TNF exposure, RIPK1 is recruited into TNFR1 and associates with an adapter protein, the TNFR1-associated death domain protein (TRADD), to form complex-I^31^. TRADD serves as an adapter for the formation of the network of polyubiquitin chains mediated by E3 ubiquitin (Ub) ligases that closely regulate the dynamic assembly of complex-I with transforming growth factor β activated kinase (TAK1) and NF-κB upstream kinase, called the IκB kinase (IKK) cascade. Although the ubiquitination of RIPK1 acts as a platform for the late cell death checkpoint by activating the NF-κB transcriptional response, it also functions to suppress the translocation of RIPK1 into cytoplasmic DISC (also known as complex-IIb), which restricts PCD by inactivating cytotoxic RIPK1^29,31^. It has been reported that RIPK1 undergoes an extensive ubiquitination process with multiple types of polyubiquitin linkages, including K63, K48, K11 and linear ubiquitination in complex-I, implying that a number of E3 ubiquitin ligases and ubiquitin cofactors are involved in this process (Table 1)^32–35^.

**Table 1:** Regulators of the cytotoxic potential of RIPK1.

Category	Regulator	Function	proposed mechanism	Outcome	Reference
E3 Ub ligases	TRAF2/5	K63-linked polyubiquitination of RIPK1 in complex-I	Preventing RIPK1 dissociation from complex-I	Inhibits complex-IIb-mediated RDA and necroptosis	^36,41,43^
	cIAP1/2	K63/K11-linked polyubiquitination of RIPK1 in complex-I	Preventing RIPK1 dissociation from complex-I	Inhibits complex-IIb-mediated RDA and necroptosis	^44–46^,
	LUBAC (HOIL, HOIP, SHARPIN)	M1-linked, linear ubiquitination of RIPK1 in complex-I	Enhanced tethering of RIPK1 in complex-I	Inhibits complex-IIb-mediated RDA and necroptosis by stabilization of complex-I	^35,50,51^
	A20	K48-linked polyubiquitination of RIPK1 in complex-I	Proteasomal degradation of RIPK1	Inhibits RDA	^74,75^
	MIB2	K63(?)-linked polyubiquitination of RIPK1 in complex-I	Inhibition of RIPK1 activity	Inhibits RDA	^64^
	PELI1	K63-linked ubiquitination of RIPK1 in complex-IIb or necrosome	promotes the interaction of RIPK1 and RIPK3	Promotes necroptosis	^140,143^
DUBs	A20	Deubiquitination of K63-Ub chains of RIPK1	Decreases RIPK1 activity by stabilizing M1-linked Ub chains	Inhibits complex-II formation and RDA and necroptosis	^74,81,82^
	CYLD	Deubiquitination of K63/M1-Ub chains of RIPK1	Decreases the overall ubiquitination status of complex-I	Promotes complex-IIb-mediated RDA and necroptosis	^89–91^
	Cezanne	Deubiquitination of K63(?)-Ub chains of RIPK1	Suppresses the build-up of polyubiquitinated RIPK1 in complex-I	promotes complex-II-mediated apoptosis	^98,99^
Adapters	ANKRD13a	UIM protein interacts with ubiquitinated RIPK1	inhibition of RIPK1 transition into complex-II	Inhibits complex-II-mediated apoptosis and necroptosis	^18^
	ABIN-1	Ub-binding protein in complex-I	Regulation of the recruitment of A20 into complex-I	Inhibits RDA and necroptosis	^62,63^
	epsin 1/2	Ub-binding endocytic adapters in complex-I	Regulation of IKKs recruitment in complex-I assembly by interacting with LUBAC	Inhibits complex-II-mediated apoptosis	^72^

Abbreviations: *RDA* RIPK1-dependent apoptosis, *RIPK1* receptor-interacting serine threonine protein kinase 1, *TRAF2/5* TNF receptor-associated factor 2/5, *cIAP1/2* cellular inhibitor of apoptosis 1/2, *LUBAC* linear ubiquitin chain assembly complex, *HOIL* heme-oxidized IRP2 ubiquitin ligase, *HOIP* HOIL-1-interacting protein, *SHARPIN* SHANK-associated RH domain-interacting protein, MIB2 Mind Bomb-2, PELI1 Pellino 1, CYLD cylindromatosis lysine 63 deubiquitinase, *Cezanne* cellular zinc finger anti-NF-kappa-B protein, *ANKRD13a* ankyrin repeat domain 13 A, *ABIN-1* A20-binding inhibitor of NF-kappaB activation 1.

TNF receptor-associated Factor 2 (TRAF2) is one of the first RING domain-containing E3 Ub ligases discovered in TNF signaling that mediates K63-linked ubiquitination of RIPK1^36^. The E3 Ub ligase activity of TRAF2 seems to be required for RIPK1 ubiquitination, as it has been observed that TNF-induced RIPK1 ubiquitination is restored by the reconstitution of wild-type TRAF2 but not by the RING domain mutant TRAF2 in TRAF2 knockout cells^37^. However, in vitro structural analysis showed that the RING domain of TRAF2 did not possess K63-linked Ub ligase activity^38,39^. Thus, it is still controversial whether TRAF2 directly ubiquitinates RIPK1 or works together with other E3 Ub ligases. At this point, one interesting observation is that murine embryonic fibroblasts (MEFs) derived from TRAF2−/− mice exhibited intact levels of RIPK1 ubiquitination and NF-κB activation in response to TNF^40^. Subsequent genetic experiments showed that TNF-induced RIPK1 ubiquitination and NF-κB activation were markedly attenuated in TRAF2 and TRAF5 double-knockout cells^41–43^, indicating that TRAF2 and TRAF5 have overlapping functions during RIPK1 ubiquitination. Thus, the exact functions of TRAF5 in mediating RIPK1 ubiquitination need to be further examined.

The cellular inhibitors of apoptosis 1 and 2 (cIAP1 and cIAP2, cIAP1/2) are also RING domain-containing E3 Ub ligases that belong to the inhibitor of apoptosis protein (IAP) family. Both of them are recruited into complex-I and play an essential role in modulating the K63-linked ubiquitination of RIPK1^44–46^. However, redundancy at this point cannot be excluded because cells bearing the cIAP1/2-interacting motif mutant of TRAF2 show reduced RIPK1 ubiquitination upon TNFR1 ligation^39^. Furthermore, RIPK1 ubiquitination is dependent on cIAP1/2 and TRAF2 interactions, but the RING domain of TRAF2 is dispensable^38,47,48^, demonstrating that TRAF2 may function to recruit cIAP1/2 in complex-I. Similarly, while TRAF2 is not always required for RIPK1 ubiquitination, data from in vitro and genetic mouse models indicate that cIAP1/2 is almost always required for RIPK1 ubiquitination and that this modification acts to prevent TNF-induced cell death^49^. Thus, genetic or pharmacological ablation of cIAP1/2 is sufficient to impair RIPK1 ubiquitination and dramatically increase the cytotoxic potential of RIPK1 via complex-IIb formation^44–46^. Consistent with the notion that cIAP1/2-mediated RIPK1 ubiquitination inhibits cell death by preventing complex-I from transitioning to complex-II, the deficiency of cIAP1 and cIAP2 leads to embryonic lethality that could be retarded by a single allele loss of RIPK1^49^.

In addition to TRAF2 and cIAP1/2, the linear ubiquitin chain assembly complex (LUBAC, consisting of the catalytic subunit HOIP, HOIL-1 and the regulatory subunit SHARPIN) was identified as an additional E3 ubiquitin ligase in complex-I^35,50,51^. In contrast to K63-linked ubiquitination by TRAF2 and cIAP1/2, LUBAC exclusively catalyzes the M1-linked linear ubiquitination of complex-I components, including TNFR1 and RIPK1^52–54^. This type of ubiquitination has a cell death suppression function similar to the K63-linked ubiquitination of RIPK1, as described above, based on observations that the suppression of HOIP or SHARPIN expression enhances complex-IIb formation and caspase-8-mediated cell death upon TNFR1 ligation in most cellular systems^55–57^. Mechanistically, LUBAC-mediated ubiquitination may delay the flux of RIPK1 to complex-IIb by tethering RIPK1 to complex-I and thus negatively regulating both apoptosis and necroptosis^58,59^. Consistent with these in vitro observations, loss of LUBAC components also results in severe inflammation and early embryonic lethality in genetic mouse models^55,60^. These results thus indicate that the M1-linked ubiquitin chain formed by LUBAC is indispensable in protecting RIPK1-mediated cell death, which functions as an important regulator of the early cell death checkpoint. Interestingly, it has been reported that the recruitment of LUBC into complex-I requires TRAF2 and cIAP1, but not RIPK1, whereas knockdown of HOIL-1 and HOIP reduces RIPK1 ubiquitination^35,51^. Thus, it is still unclear whether RIPK1 is a direct substrate for the E3 ubiquitin ligase activity of LUBAC, and the precise mechanisms and ubiquitination sites on RIPK1 for LUBAC remain to be further elucidated. In this regard, it has been reported that ABIN-1 (A20-binding inhibitor of NF-κB activation 1), a ubiquitin-binding protein, can interact with both M1-linked and K63-linked ubiquitin chains^61^. Further studies have revealed that the recruitment of ABIN-1 into complex-I by association with LUBAC and cIAP1/2 leads to the suppression of RIPK1-dependent apoptosis and necroptosis^62,63^. It is thus hypothesized that ABIN-1 functions as a critical link between M1/K63-linked ubiquitination to control the cytotoxic activity of RIPK1 by LUBAC.

Mind Bomb-2 (MIB2) is another E3 Ub ligase that has been reported to regulate the cytotoxic potential of RIPK1^64^. In this study, MIB2 was capable of inducing the K63-linked ubiquitination of RIPK1 at different sites, including K115 and K377, and such ubiquitination repressed the kinase activity of RIPK1^64^. Consequently, depletion of MIB2 sensitized cells to RIP1-dependent cell death via increased complex-II formation^64^. Thus, these data support a role for MIB2 as an additional early cell death checkpoint regulator. However, this study also questioned the functional relevance of K63-linked ubiquitination of RIPK1 in NF-κB signaling based on observations; depletion of MIB2 has little effect on NF-kB activation after TNF stimulation^64^.

Previously, it was reported that internalization of the TNFR1 signaling complex via endocytic trafficking plays an important role in complex-IIb formation in nonphagocytic cells^65–68^. In addition, the ubiquitin interacting motif (UIM) is found in proteins either involved in ubiquitination or in those known to interact with ubiquitin-like modifiers that function to regulate TNFR1 internalization and its cellular trafficking^69,70^. Recently, it has been reported that ankyrin repeat domain 13a (ANKRD13a), which carries multiple copies of UIM, interacts with ubiquitinated RIPK1 upon TNFR1 ligation, which sets a higher signal threshold for the cytotoxic potential of RIPK1: loss of AKRD13a can switch TNF signaling from survival to death by promoting the formation of complex-IIb^71^. However, because ANKRD13a is not an E3 Ub ligase itself, it is unclear how ANKRD13 negatively regulates the cytotoxic potential of RIPK1. Intriguingly, epsin proteins (epsin 1 or epsin 2, a family of ubiquitin-binding endocytic adapters) were found to be adapter proteins that interact with the components of complex-I, including RIPK1, HOIL-1 and HOIP, in a UIM-dependent manner^72^. These findings thus indicate that UIM-containing adapter proteins such as ANKRD13a and epsin 1/2 are involved in TNF signaling by keeping the specific endocytic assembly in check to inhibit the RIPK1 transition to complex-IIb, thereby reducing RIPK1 cytotoxicity. Further structural analyses via spatial compartmentalization and via integrated networks of these UIM-containing proteins in complex-IIb are necessary to elucidate the precise mechanisms by which UIM-containing adapter proteins limit the cytotoxic potential of RIPK1.

## Regulation of the cytotoxic potential of RIPK1 by deubiquitination in complex-I

In addition to investigations on RIPK1 ubiquitination-mediated E3 Ub ligases, as discussed earlier, substantial progress has been made in understanding the role of RIPK1 deubiquitination regulated by multiple deubiquitinating enzymes (DUBs), including A20, CYLD and Cezanne.

The zinc finger protein A20 (also known as TNFAIP3) plays an essential role in limiting inflammatory responses by the negative feedback regulation of NF-κB signaling^73^. As one of the early NF-κB response genes, the expression of A20 is induced by TNF and recruited into TNFR1 complex-I and acts as a dual ubiquitin editing enzyme on RIPK1^73^. Once A20 is recruited into complex-I, the N-terminal ovarian tumor (OTU) domain functions as a DUB that removes K63-linked polyUb chains from RIPK1 to prevent excessive NF-κB activation^74,75^. Interestingly, the C-terminal domain of A20, composed of seven zinc fingers, has been shown to function as an E3 Ub ligase that mediates K48-linked ubiquitination of RIPK1, thereby targeting its proteasomal degradation^74^, suggesting that A20 restricts the accessibility of IKKs into complex-I via its dual Ub editing activity of RIPK1. Accordingly, A20-deficient cells exhibit severe defects in the termination of TNF-induced NF-κB activation via aberrant ubiquitination of RIPK1 in complex-I^76^. Given the ability of A20 to terminate NF-κB activity, A20 is expected to possess a pro-cell death function. However, a growing body of evidence indicates that A20 protects most cells from death receptor-mediated cell death^77,78^. In fact, A20-deficient mice show premature death phenotypes due to severe inflammation^76,79^. Based on these observations, it seems that A20 likely exerts its anti-cell death function independently of its DUB activity. However, it has been reported that A20 is recruited into complex-I via LUBAC-mediated linear ubiquitination^80^. Even though A20 is unable to function as a DUB to cleave M1-linked linear ubiquitination, A20-deficient cells were found to have markedly decreased M1 ubiquitination of RIPK1^81^, consistent with the results that A20 stabilizes the M1-linked ubiquitin chains in complex-I^82^. The cumulative evidence suggests that A20 plays a role in regulating the early cell death checkpoint during RIPK1-mediated cell death.

CYLD was initially identified as a tumor suppressor mutated in cylindromatosis characterized by a predisposition toward forming benign tumors of skin appendages^83,84^. Further in vitro studies revealed that CYLD functions as a DUB by removing K63 ubiquitin chains from various target proteins, including RIPK1, TRAF2, and NEMO/IKK-γ^85–88^. Consistent with the well-established DUB function, CYLD promotes formation of the cytosolic RIPK1 signaling complex, including either complex-II or necrosome, which elicits the RIPK1-dependent dual modes of PCD^81,89–91^. Importantly, genetic deletion of CYLD in mice results in the accumulation of ubiquitinated RIPK1, which is associated with the attenuation of the early wave of germ cell apoptosis^92^, suggesting that the DUB function of CYLD preferentially targets RIPK1 rather than TRAF2 or NEMO/IKK-γ to promote RIPK1-dependent cell death. While A20 hydrolyzes K63 and K48, but not M1 ubiquitination linkages, CYLD hydrolyzes K63 and M1 ubiquitination linkages induced by cIAP1/2 and LUBAC, respectively^81^. The removal of LUBAC-mediated M1 ubiquitination linkages by CYLD further modifies the overall ubiquitination status of complex-I to facilitate RIPK1-driven cytotoxicity^81,89^. Consistent with in vitro observations, reduced levels of CYLD expression have been reported in various human cancer tissues^93,94^. Accordingly, genetic studies have shown that CYLD is frequently inactivated by somatic mutations in many cancers, including hepatocellular carcinoma, multiple myeloma, and head and neck cancer^95–97^. In this regard, it is likely that s CYLD mutation or deficiency in some cancers decreases its deubiquitination activity against K63-linked or M1-linked Ub chains, leading to insufficient activation of RIPK1-mediated cell death.

In addition, it has been reported that Cezanne, a member of the A20 family of deubiquitinating cysteine proteases, also functions as a DUB on RIPK1^98^. Cezanne can be induced following TNF exposure and is recruited into complex-I, where it cleaves polyubiquitin chains from RIPK1^99,100^, suggesting that both Cezanne and A20 are endogenous negative regulators of the NF-κB signaling pathway by a negative feedback loop to prevent excessive activation at the level of complex-I. This deubiquitinating property of Cezanne against RIPK1 supports its role in regulating RIPK1-mediated cell death. However, since Cezanne and A20 possess overlapping biochemical properties, further characterization of divergent specificities toward other TNFR1 signaling components is required to provide an explanation for their redundant functions.

## Regulation of the cytotoxic potential of RIPK1 by autophosphorylation

The kinase activity of RIPK1 is required for the apoptosis induced by RIPK1 deubiquitination. Loss of the E3 Ub ligases cIAP1 and cIAP2 in TNF-treated cells is sufficient to induce the formation of death-inducing complex-IIb containing RIPK1, FADD and caspase-8 to drive RIPK1 activation^101–103^. Such results suggest that the ubiquitination and phosphorylation processes of RIPK1 are closely interlinked. Furthermore, it has also been reported that genotoxic stress-mediated depletion of cIAP1 and cIAP2 causes spontaneous association of RIPK1/FADD/caspase-8 in the cytosol even in the absence of TNFR1 ligation^104,105^. This TNFR1-independent RIPK1-containing cytosolic platform has been referred to as the Ripoptosome, and it relies on RIPK1 kinase activity that can cause either apoptosis or necroptosis, depending on the cellular context^106^. However, how deubiquitination of RIPK1 affects its kinase activity in complex-IIb or Ripoptosome remains largely unresolved. Ectopic expression of RIPK1 is able to induce the dimerization and/or oligomerization of RIPK1 via its death domain^107^. Furthermore, autophosphorylation at S161 and S166 of RIPK1 induces conformational changes that allow RIPK1 to interact with death effectors such as FADD and RIPK3, thereby facilitating the formation of complex-IIb or necrosomes for the induction of apoptosis or necroptosis, respectively^108–110^. Such observations suggest that the transition of RIPK1 into cytosolic complex-IIb as a result of ubiquitination may result in its oligomerization in either complex-II or necrosomes, leading to its autophosphorylation at serines 161 and 166. Notably, reconstitution of the RIPK1-S161A mutant in RIPK1-deficient cells did not affect TNF-induced necroptosis^111^. Importantly, recent studies using mice containing an S166A RIPK1 knock-in allele showed that S166 phosphorylation plays a critical role in triggering RIPK1 activation but is insufficient to trigger RIPK1-mediated cell death^108^. Thus, it is possible that RIPK1 S166 phosphorylation permits the autophosphorylation of additional sites on RIPK1 to drive RIPK1-mediated PCD. These findings provide experimental evidence that RIPK1 autophosphorylation at S166 can serve as a biomarker of RIPK1 kinase activity.

Reactive oxygen species (ROS) have long been considered a driving force for RIPK1-dependent necroptosis, but a mechanistic understanding has been lacking^9,112,113^. Recent studies have revealed that the enhanced levels of ROS induced by TNF or some chemotherapeutic agents act as a positive feedback loop to enhance necrosome formation via RIPK1 autophosphorylation at Ser161^114–116^. Mechanistically, ROS are capable of promoting RIPK1 autophosphorylation at Ser161, which promotes RIPK1 oligomerization and RIPK1/RIPK3 interactions^116^. This suggests that ROS induce a positive feedback loop during necrosome formation by targeting RIPK1. However, ROS production during necroptosis also occurs downstream of RIPK1^9^; thus, the signaling pathways governing the crosstalk between ROS and RIPK1 in the course of necroptosis require further investigation.

## Regulation of the cytotoxic potential of RIPK1 by transphosphorylation

**Fig. 2:**
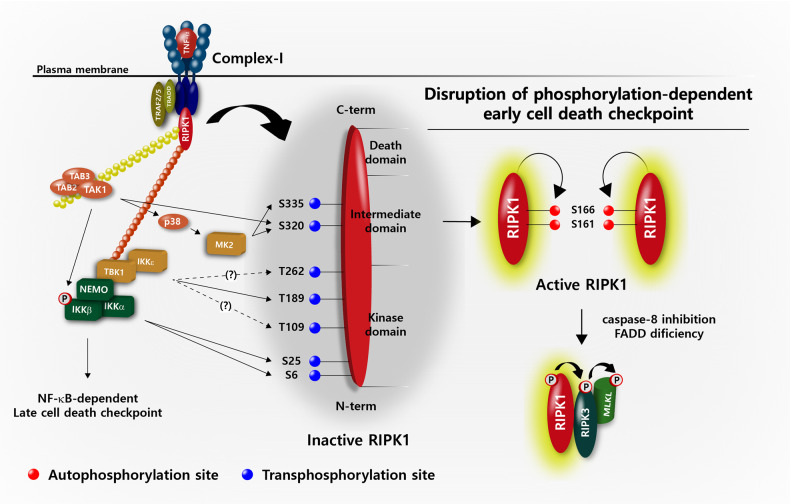
Regulation of RIPK1 cytotoxicity by phosphorylation. Upon TNFR1 ligation, RIPK1 is recruited to membrane-associated complex-I and is ubiquitinated and then functions as a scaffold to interact with IKK and its related kinases that play essential roles in NF-κB and p38 MAPK activation. In addition to the role of the upstream kinases for NF-κB signaling, TAK1 and IKKs directly phosphorylate the ubiquitinated RIPK1 bound to complex-I, which suppresses the cytotoxic potential of RIPK1 via an unknown mechanism, possibly by preventing the translocation of RIPK1 into complex-IIb. In addition to TAK1 and IKKs, TBK1/IKKε and MK2 also inhibit the full activation of RIPK1 via direct phosphorylation of RIPK1 that is bound to complex-I and of the cytosolic pool of RIPK1, respectively. If the phosphorylation-mediated early cell death checkpoint is disrupted by inactivation of these kinases, RIPK1 is autophosphorylated via an allosteric effect and switches to an active death-inducing mode where it subsequently phosphorylates necrosome-associated RIPK3 to initiate necroptosis. The key kinases that phosphorylate RIPK1 at distinct sites are indicated. A broken arrow indicates indirect phosphorylation sites on RIPK1 for TBK1/IKKε. Abbreviations: TNFR1 tumor necrosis factor receptor 1, RIPK1/3 receptor-interacting serine threonine protein kinase 1/3, MAPK mitogen-activated protein kinase, TAK1 transforming growth factor β activated kinase 1, IKK inhibitory kappa B kinase, TBK1/IKKε TANK-binding kinase 1/IKK-epsilon, MK2 MAPK-activated protein kinase 2.

As described above, the autophosphorylation of RIPK1 at Ser 161 and S166 in complex-II functions as a positive regulator of the kinase activity of RIPK1. In contrast, recent studies have revealed that phosphorylation of RIPK1 by several kinases in complex-I acts as a fine-tuning negative mechanism to repress RIPK1 activation^26,27,117^ (Fig. 2).

Upon TNFR1 ligation, TAK1 and IKKs (IKK-α/-β/-γ) are recruited into complex-I by interacting with ubiquitinated RIPK1, which functions as an upstream kinase for NF-κB-dependent prosurvival functions^118^. While activation of TAK1 and/or IKKs is dependent on their ability to interact with ubiquitinated RIPK1, they also act at the early cell death checkpoint to repress the cytotoxic potential of RIPK1 through phosphorylation^25,119^. Researchers at the Bertrand group made the unexpected observation that inhibition of IKKα or IKKβ activity induces RIPK1-dependent apoptosis or necroptosis by promoting the formation of complex-IIb or necrosomes in an NF-κB-independent manner^120^. In line with these findings, both in vitro and in vivo studies showed that inhibiting IKKβ activation results in excessive RIPK1 phosphorylation and RIPK1-mediated necroptosis^27,121^. Subsequent work has revealed that IKKα and/or IKKβ directly phosphorylate RIPK1 at Ser25 in complex-I, leading to suppression of RIPK1 kinase activity and its autophosphorylation^122^. Thus, IKKα/IKKβ appears to function as an early checkpoint to shut down the pro-death activity of RIPK1. However, reconstitution of a phospho-deficient alanine mutant (S25A) of RIPK1 in RIPK1-deficient cells is not sufficient to completely prevent RIPK1-mediated PCD upon TNFR1 ligation^122^. This finding indicates that, in addition to Ser25, IKKα/IKKβ might phosphorylate other sites at RIPK1. While proteomic studies using mass spectrometric analysis have revealed the existence of many additional residues of RIPK1 that are phosphorylated, such as Ser25, Ser166, S331 and Ser416^120,122^, the functional consequence of these phosphorylation events has not been examined. Furthermore, it is also unclear whether the phosphorylation of RIPK1 by IKKα/IKKβ directly affects the recruitment of RIPK1 into complex-IIb or prevents the dissociation of RIPK1 from complex-I. Apart from IKKα/IKKβ, the noncanonical IKK-related kinases TANK-binding kinase 1 (TBK1) and IKKε have recently been reported to phosphorylate RIPK1 in complex-I, and this phosphorylation prevents RIPK1-dependent PCD^123,124^. Similar to IKKα/IKKβ, the recruitment of these kinases into complex-I depends on RIPK1 and its K63-linked and linear ubiquitination by cIAP1/2 and LUBAC^124^. However, in contrast to IKKα/IKKβ, TBK1 and IKKε do not function as upstream kinases of the canonical NF-κB activation pathway^125^, indicating that they solely limit RIPK1 cytotoxicity by preventing the formation of complex-IIb or necrosomes without affecting the transcription-dependent late cell death checkpoint. Accordingly, TBK1 is able to directly phosphorylate human RIPK1 at T189 (equivalent to T190 mouse RIPK1) to regulate the cytotoxic potential of RIPK1^123,124^. Since inhibition of either IKKα/IKKβ or TBK1/IKKε is sufficient to trigger RIPK1-dependent PCD upon TNFR1 ligation, further work is required to elucidate whether their respective phosphorylation sites in RIPK1 have any redundancy in regulating RIPK1 cytotoxicity.

In addition to IKKs, activation of TAK1 also functions as an important negative regulator of RIPK1 cytotoxicity through phosphorylation. Genetic deletion of the pharmacological inhibition of TAK1 elicits RIPK1-mediated PCD without affecting RIPK1 ubiquitination in complex-I, in which TAK1 acts to repress RIPK1 autophosphorylation, independent of IKK-mediated NF-κB activation^120,126^. In line with this, a TAK1 deficiency not only sensitized cells to TNF-induced PCD but also enhanced the interaction between RIPK1 and RIPK3 under the conditions of a caspase-8 blockade^116,117^. Thus, activation of RIPK1 cytotoxicity by manipulation of its upstream kinase activities, such as those of TAK1 and IKKs, may provide new therapeutic options for TNF-mediated diseases. In fact, small molecular inhibitors of TAK1 or IKKβ are capable of inducing RDA in a TNF-dependent manner in some cancer and autoimmune disease models by dysregulating RIPK1 phosphorylation^127,128^. Recently, it was reported that S320 in human RIPK1 (equivalent to S321 in mouse RIPK1) is phosphorylated by TAK1 and thus attenuates RIPK1 cytotoxicity^129^. Accordingly, loss of S320 phosphorylation of RIPK1 drives RIPK1-mediated PCD by facilitating the formation of complex-II and necroptosis under NF-κB defective conditions^129^. Importantly, in cIAP1/2 double knockout cells, RIPK1-S320 phosphorylation following TNF treatment was almost completely negated^129^, suggesting that the K63-linked ubiquitination of RIPK1 mediated by cIAP1/2 may promote recruitment of TAK1 into complex-I. Further work is needed to accurately define the role of specific types of ubiquitin conjugation events in regulating RIPK1 phosphorylation mediated by TAK1.

MAPK-activated protein kinase 2 (MK2), a kinase downstream of p38 MAPK, was initially identified as a downstream effector of TNFR1 since it is essential for TNF biosynthesis via posttranscriptional regulation^130,131^. Unexpectedly, when studying the therapeutic potential of the cIAP1/2 inhibitor (smac mimetics; SM) in a leukemia model, it was noticed that MK2 inhibitors sensitized SM-induced cytotoxicity in a RIPK1-dependent manner^132^. Furthermore, it was observed that MK2 knockout mice showed enhanced cytotoxic sensitivity to TNF administration^133^. These perplexing observations led to the idea that the p38-MK2 axis might have an additional role in regulating cell death signaling downstream of TNFR1. In fact, this notion is supported by subsequent observations that MK2 phosphorylates human RIPK1 at Ser 320 and S335 (equivalent to S321 and S335 in mouse RIPK1) to inhibit RIPK1 cytotoxicity in TNFR1 signaling^34,134,135^. Thus, it appears that MK2 plays a role in limiting TNF cytotoxicity in the timely and coordinated TNFR1 complex, independent of the generation of ligands, including TNF. Further studies of the in vivo physiological significance of this dual action of MK2 could provide new perspectives for understanding TNF-driven cell death and inflammation. Since the phosphorylation of RIPK1 at different serine residues by IKKs and MK2 simultaneously provides a prosurvival signal by limiting RIPK1 cytotoxicity, the functional implication of multiple RIPK1 phosphorylation events by its upstream kinases remains to be further elucidated. In this regard, MK2 inhibition selectively enhances RIPK1-mediated cell death during TNF signaling when IKKs or cIAP1/2 is inhibited or depleted^34,134^. Mechanistically, RIPK1 phosphorylation by MK2 negatively regulates complex-IIb-mediated cell death by suppressing RIPK1 autophosphorylation^34,134^. More importantly, the current body of knowledge seems to suggest that IKKs and MK2 may target spatially different pools of RIPK1 for phosphorylation. IKKs are recruited into complex-I and phosphorylate plasma membrane-bound RIPK1 in a ubiquitination-dependent manner^25,119^. In contrast, MK2 mainly phosphorylates the cytosolic pool of RIPK1, which might be subsequently recruited into complex-I^34^. Thus, it is possible that IKK and MK2 provide separate early cell death checkpoints for RIPK1 activation by phosphorylating spatially distinct RIPK1 pools to limit the full activation of the cytotoxic potential of RIPK1^136,137^. However, given that complex-IIb, which contains RIPK1, is sequentially dissociated from complex-I^26,138^, the primary source of RIPK1 bound to death-inducing complex-IIb is still a longstanding issue to clarify.

## Regulation of the cytotoxic potential of complex-IIb/necrosome-associated RIPK1

Given that deubiquitination of RIPK1 is required for its transition from complex-I to complex-IIb^29,31^, it is believed that the RIPK1 tethered within complex-I is more heavily modified with diverse polyubiquitin chains than the RIPK1 tethered within complex-IIb or necrosomes. However, several pieces of evidence have suggested that necrosome-associated RIPK1 also undergoes ubiquitination during necroptosis. For instance, although CYLD functions as a DUB for RIPK1 in complex-I^81^, ubiquitin-like modifications of RIPK1 were observed in detergent-insoluble cytosolic fractions^11^, and a CYLD deficiency led to hyperubiquitinated RIPK1 in the necrosome, which impaired the formation of the RIPK1-RIPK3 complex^89^. These observations raise the possibility that the ubiquitination of RIPK1 in spatially distinct necrosomes may directly control its cytotoxicity. Accordingly, it has recently been reported that necrosome-associated RIPK1 undergoes K63- and M1-linked polyubiquitination by cIAP1/2 and LUBAC, respectively^52,139^, but the functional link between these E3 Ub ligases and RIPK1 cytotoxicity during the necroptosis signaling pathway is less understood. Recent studies have proposed a possible role for specific ubiquitination sites of RIPK1 at K115 that function as positive regulators for necroptosis by facilitating RIPK1-RIPK3 interactions^139^. Interestingly, mutation of this ubiquitination site on RIPK1 (K115) suppressed RIPK1 autophosphorylation and necroptosis without affecting the inherent RIPK1 kinase activity^139^, indicating that it may also play an additional role in determining cell death by modifying the ubiquitination status of necrosome-associated RIPK1. Nevertheless, since RIPK1 ubiquitination occurs in the necrosome complex independent of RIPK3 and MLKL^139^, the question remains whether the ubiquitinated RIPK1 at K115 found in necrosomes was translocated from complex-IIb. A member of the Pellino family, Pellino 1 (PELI1), an E3 Ub ligase, may provide the possibility for ubiquitination of RIPK1 in complex-IIb^140–142^. PELI1 is not involved in RIPK1 ubiquitination during TNF-induced apoptosis, but it modulates K63-linked ubiquitination of RIPK1 on K115 bound to complex-IIb in a kinase-dependent manner that promotes the interaction of RIPK1 and RIPK3 to form necrosomes^140^. Thus, PELI1 can function as a pronecroptotic E3 Ub ligase that targets kinase-active RIPK1 in complex-IIb. Paradoxically, PELI1 was reported to exhibit a protective effect in the context of necroptosis by regulating the protein level of RIPK3^143,144^. PELI1 directly catalyzes K48-linked ubiquitination of RIPK3 for proteasomal degradation and thus selectively restricts RIPK3-mediated necroptosis^144^. Thus, this finding for PELI1 in the context of necroptosis execution is still under debate^140,143,144^. Since RIPK3 kinase activity is required for PELI1 recruitment^145^, it is possible that PELI1 differentially targets RIPK1 or RIPK3 depending on the status of RIPK3 activation. Further in vitro biochemical and in vivo animal experiments are required to confirm the pathophysiological pathway by which PELI1 regulates PCD in complex-IIb or necrosomes by RIPK1 or RIPK3.

## Clinical relevance of RIPK1 cytotoxicity in inflammation and cancer immunotherapy

Unlike apoptosis, necroptosis is defined as an inflammatory form of cell death induced by the release of damage-associated molecular patterns (DAMPs) and aberrant inflammatory cytokines in dying cells^146^. Given the pivotal role of the kinase activity of RIPK1 in controlling necroptosis, RIPK1-mediated cell death is involved in a variety of pathophysiological settings, including inflammation and viral and bacterial infections^147^. Thus, regulation of the early cell death checkpoint that safeguards the cytotoxic potential of RIPK1 is of clinical relevance, potentially offering new therapeutic opportunities for chronic inflammatory diseases, such as rheumatoid arthritis, psoriasis and septic shock^145,148,149^. Consistent with this notion, GSK2982772, a highly specific RIPK1 inhibitor, has been developed^150^ and has successfully completed phase II of human clinical trials for the treatment of ulcerative colitis (NCT02903966), psoriasis (NCT02776033) and rheumatoid arthritis (NCT02858492), with an excellent safety profile^151,152^. Moreover, families of allosteric RIPK1 inhibitors, DNL758/788 (NCT03757325) and R552 (NCT03757351), are currently undergoing phase Ia/Ib or IIa clinical trials for various neuro-inflammatory diseases, including cutaneous lupus erythematosus, multiple sclerosis, Alzheimer’s disease and amyotrophic lateral sclerosis^153^.

Since unresolved inflammation is necessary for cancer progression^154,155^, it is believed that an aberrant innate and adaptive immune response contributes to tumorigenesis by inducing immune suppression and favoring aggressive clones for recurrence and/or metastasis^156^. While inhibition of RIPK1 activation has therapeutic potential for treating the abovementioned inflammatory or autoimmune diseases, the inflammatory nature of RIPK1-mediated cell death promotes immunogenicity and macrophage-mediated adaptive immune tolerance in some cancers^157^. Consistent with this notion, patients with severe immune deficiency, inflammatory bowel disease and arthritis have biallelic mutations of RIPK1^158,159^. Mechanistically, activation of RIPK1 and NF-κB signaling in dying cells stimulates immune responses, which subsequently drive dendritic cell (DC) maturation for CD8^+^ T-cell cross-priming^160–162^. Consequently, ectopic injection of RIPK1-derived necroptotic cells into the tumor microenvironment induces a systemic immune response driven by DCs and CD8^+^ T cells that promotes antitumor immunity and increased tumor antigen loading^163^. Thus, manipulating RIPK1 checkpoints to boost its cytotoxicity in cancer cells might be an attractive therapeutic avenue in which immunogenic cell death is a desired outcome.

Given that RIPK1 ubiquitination can safeguard the killing potential of RIPK1, small molecular mimetics of smac/DIABLO (smac-mimetics; SMs) have been developed to disrupt RIPK1 ubiquitination by antagonizing cIAP1/2^164–166^. At present, these SMs have shown promising therapeutic potential for cancer^167,168^. In addition to killing cancer cells, a number of studies have also reported the effects of SMs on both the innate and adaptive immune systems during cancer^169^. In some instances, SM administration not only induces a reversion of tumor-associated macrophages from a protumorous M2 phenotype to a proinflammatory M1-like phenotype^170^ but also sensitizes resistant cancer cells to inactivate by an inflammatory burst of TNF and IFN-γ^171–173^. Accordingly, several SMs, such as LCL161 and Debio1143, are undergoing initial clinical trials (http://clinicaltrials.gov). One of the most clinically advanced SMs (birinapant) is currently undergoing phase I/II clinical trials for a number of cancers, including myeloid leukemia and various solid tumors^174–176^. However, despite the promising preclinical results, the clinical trials have largely failed due to their poor efficacy when used as individual agents^168,177^. Thus, the challenge for future immunotherapy lies in research efforts directed toward the use of SMs as part of combination strategies to overcome resistance. In this regard, the recent discoveries of RIPK1-mediated early cell death checkpoints are of particular interest, especially with respect to combination strategies that incorporate SMs. In fact, activating the RIPK1-mediated cell death checkpoint by clinical inhibitors IKK or MK2 enhances the therapeutic efficacy of birinapant by triggering immunogenic cell death and antigen-cross priming CD8^+^ T cells in primary acute myeloid leukemia^132,178,179^. While many studies have demonstrated that necroptosis-mediated inflammatory cell death can promote antitumor immune responses^163,180,181^, it has also been reported that RIPK1 activity regulates tumor immunity by reprogramming tumor-associated macrophages in some cancers, independent of RIPK3^157,182^. At this point, there is still a limited understanding of the specific involvement of RIPK1 in the tumor immune microenvironment. Nevertheless, a better understanding of the mechanism that regulates the cytotoxic activity of RIPK1 in immune cells may offer new therapeutic options for chronic inflammatory diseases and cancer.

## Conclusions and perspectives

While RIPK1 was originally characterized as a modulator of NF-κB activation through its interaction with IKKs, it is now clear that RIPK1 functions as a crucial early cell death checkpoint for determining the fate of cells, and this function is mainly regulated via its PTMs, including ubiquitination and phosphorylation. Considering the necessity of the kinase activity of RIPK1 for driving the necroptotic mode of cell death, the RIPK1-mediated cell death checkpoint could be targeted therapeutically to modulate human inflammatory diseases and cancer. In this sense, it will be important to identify pharmacological agents that directly control RIPK1 cytotoxicity to overcome some of the challenges associated with conventional immunotherapies. However, to date, the exact molecular mechanisms regulating RIPK1 ubiquitination or phosphorylation and its cytotoxic potential remain to be fully elucidated. Furthermore, it is still not clear whether ubiquitinated RIPK1 in necrosomes originates from the plasma membrane-bound complex-I or from the cytosolic pool. Thus, the compositions of the RIPK1-containing complex in complex-IIb and necrosomes and their subcellular localizations require further investigation. Studies addressing this issue would help our understanding of the molecular mechanisms that control the formation of death-inducing signaling complexes or the cytotoxic potential of RIPK1. Finally, the extra layer of complexity involved in regulating RIPK1 activity presents challenges, especially for the development of therapeutic agents that target the RIPK1-mediated cell death checkpoint for clinical applications in human diseases in the necroptosis era.
